# How Ambivalence Toward Digital–AI Transformation Affects Taking-Charge Behavior: A Threat–Rigidity Theoretical Perspective

**DOI:** 10.3390/bs15030261

**Published:** 2025-02-24

**Authors:** Xueliang Pei, Jianing Guo, Tung-Ju Wu

**Affiliations:** 1 College of Business Administration, Huaqiao University, Quanzhou 362021, China; peixueliang@hqu.edu.cn; 2 East Business Management Research Centre, Huaqiao University, Quanzhou 362021, China; 3 School of Management, Beijing Institute of Technology, Beijing 100081, China; jnine7681@163.com; 4 School of Management, Harbin Institute of Technology (HIT), Harbin 150001, China

**Keywords:** ambivalence toward digital–AI transformation, taking-charge behavior, threat–rigidity theory

## Abstract

Digital–AI transformation is revolutionizing the modern workplace, yet its complexity has left many aspects of employee responses underexplored. While previous research has examined some employee reactions to technological change, the nuanced impact of ambivalence toward digital–AI transformation on employees’ proactive behavior remains a largely uncharted area. This is especially significant as proactive behavior is crucial for the successful implementation of digital–AI transformation. While presenting unprecedented opportunities, digital–AI transformation has also triggered intricate psychological responses among employees, with ambivalence toward it being particularly prominent. Building on threat–rigidity theory, this study aims to fill a research gap by exploring the impact of ambivalence on employees’ proactive behavior during digital–AI transformation. Using survey data collected from 343 employees undergoing digital–AI transformation, we tested a structural model linking ambivalence, job engagement, and future work self-salience to taking-charge behavior. The results reveal that ambivalence toward digital–AI transformation negatively affects taking-charge behavior. Furthermore, both future work self-salience and job engagement fully mediate this relationship. Additionally, job engagement and future work self-salience jointly play a chained mediating role in the negative effect of ambivalence toward digital–AI transformation on taking-charge behavior. Our findings provide actionable insights for organizations seeking to mitigate ambivalence and foster proactive employee engagement in digital transformation initiatives.

## 1. Introduction

In the contemporary era, a new wave of technological change is surging, driven by digital intelligence technologies like AI, algorithms, big data, and cloud computing (N. Chen et al., 2024; Wu et al., 2024). This wave is sweeping across various industry sectors with unprecedented influence. In response, many organizations have initiated digital–AI transformation to boost efficiency and competitiveness (Suchman, 2007). However, the success of this transformation relies not only on technology. Since technology has social properties and people are at the forefront of digital transformation, an organization’s workforce is crucial (Suchman, 2007). Overlooking employees’ reactions to digital–AI transformation, such as their awareness of technological change, can have negative effects. These effects can hinder personal growth and organizational transformation and risk long-term development (Trenerry et al., 2021; Westerman, 2016). Thus, enterprises must consider employees’ reactions from a human-centered perspective.

While prior studies have explored employees’ reactions to digital–AI transformation, they have predominantly adopted a binary perspective—focusing on either positive or negative attitudes (Wu et al., 2024; Oreg & Sverdlik, 2011). However, employees often experience mixed feelings leading to ambivalence, defined as coexisting positive and negative reactions toward a specific object (Kaplan, 1972; van Harreveld et al., 2009). Although ambivalence has been linked to outcomes such as creativity and adaptive performance (Zhang et al., 2022; Vakola et al., 2021), its behavioral consequences in digital–AI transformation remain underexplored. For instance, while Zhang et al. (2022) examined how ambivalent leadership fosters creativity, and Vakola et al. (2021) highlighted work engagement as a mitigator of ambivalence during organizational change, these studies do not address how ambivalence itself shapes proactive behaviors critical to digital–AI success. This gap is problematic: the lack of integration between positive and negative perspectives risks misinterpretations of employee reactions and undermines practical efforts to manage change (Wu et al., 2024; Oreg & Sverdlik, 2011). By integrating both perspectives and examining their combined effects on proactive behavior, this study aims to address this critical gap, offering a more nuanced understanding of how ambivalence influences employees in digital–AI transformation contexts.

To address this gap, this study focuses on a key behavioral outcome in digital–AI transformation: taking-charge behavior. As organizations navigate digital–AI transformation, they face unprecedented challenges driven by external competition and internal change demands. Leaders, constrained by limited skills and energy, increasingly rely on employees to not only perform routine tasks but also initiate change. Employees are expected to break traditional work patterns and explore new, efficient approaches aligned with digital–AI transformation (Morrison & Phelps, 1999; Grant et al., 2009). Such proactive behaviors, termed taking charge, are critical for organizational innovation, adaptability, and employee satisfaction (Kim & Liu, 2017). Thus, this study seeks to answer the following questions: How does ambivalence toward digital–AI transformation influence taking-charge behavior, and what psychological mechanisms mediate this relationship? In this way, we aim to provides theoretical insights and practical guidance for organizations to better support employees during digital–AI transformation.

In exploring how ambivalence influences taking-charge behavior, we draw on threat–rigidity theory (Staw et al., 1981; Van Hootegem et al., 2019). This theory posits that perceived threats trigger cognitive and behavioral rigidity, reducing individuals’ willingness to engage in proactive or innovative actions. Ambivalence, by its very nature, creates psychological tension and uncertainty (Kaplan, 1972; van Harreveld et al., 2009), as employees simultaneously perceive digital–AI transformation as both an opportunity and a threat. This tension amplifies their sense of risk, as they struggle to reconcile conflicting emotions and anticipate potential negative outcomes (Staw et al., 1981). For example, employees may fear job displacement due to automation while also recognizing the potential for skill development and career growth. Such conflicting perceptions heighten their subjective sense of threat, leading to cognitive rigidity—a narrowed focus on avoiding losses rather than pursuing gains (Van Hootegem et al., 2019; Muurlink et al., 2012).

Despite limited empirical research on threat–rigidity theory (Van Hootegem et al., 2019), its theoretical core offers a robust framework to explain the link between ambivalence and taking-charge behavior in digital–AI transformation. The theory highlights that psychological stress, triggered by perceived threats, manifests as cognitive and behavioral rigidity (Staw et al., 1981), providing a unique framework for analyzing how ambivalence influences employees’ proactive behaviors. Specifically, we propose that ambivalence operates through two key pathways—job engagement and future work self-salience—to induce behavioral rigidity, ultimately reducing employees’ willingness to take charge. On the one hand, job engagement reflects a psychological state of high attention and strong connection to one’s work (Kahn, 1992), enabling employees to expand their roles and adopt proactive behaviors. However, ambivalence undermines engagement by creating psychological tension and diverting cognitive resources toward managing conflicting emotions. For example, employees torn between the opportunities and threats of digital–AI transformation may struggle to fully immerse themselves in their work, leading to reduced initiative and innovation (Kahn, 1992). On the other hand, future work self-salience captures employees’ aspirations for an idealized professional identity (Strauss et al., 2012), serving as a motivational compass that helps them overcome self-doubt and career uncertainty. When future work self-salience is high, employees are more likely to engage in proactive career behaviors, such as skill development and organizational change (Strauss et al., 2012; Arif et al., 2017), yet ambivalence weakens this mechanism by amplifying uncertainty about the future. For instance, employees uncertain about how digital–AI transformation will impact their careers may lose sight of their professional goals, reducing their willingness to take charge (Strauss et al., 2012).

Critically, future work self-salience and job engagement are interconnected. Research suggests that future work self-salience enhances job engagement by providing employees with a sense of direction and purpose (W. Lin et al., 2016). When employees have a clear vision of their future, they are more likely to invest cognitively and emotionally in their current work. Conversely, when ambivalence clouds their future aspirations, it not only diminishes future work self-salience but also erodes job engagement, creating a cycle of reduced proactive behaviors. Building on this, we examine the chain mediation effect of future work self-salience and job engagement, offering a nuanced understanding of how ambivalence influences taking-charge behavior in digital–AI transformation contexts.

This study contributes to the literature in three key ways. First, it integrates both positive and negative reactions to digital–AI transformation, offering a holistic view of ambivalence and its dual nature. Second, it advances understanding of how ambivalence translates into behavioral outcomes by anchoring its investigation in threat–rigidity theory (Staw et al., 1981; Van Hootegem et al., 2019), which posits that perceived threats trigger cognitive and behavioral rigidity. Third, it introduces a psychological mechanism—job engagement and future work self-salience—to explain how ambivalence influences proactive workplace behavior. In this way, this study not only addresses a critical gap in the literature but also provides actionable insights for organizations navigating digital–AI transformation.

The rest of this study is organized as follows: the Section 2 contains the theoretical background and hypothesis development. The Section 3 describes the method, specifically including the measurement instruments as well as the participants and procedures. The Section 4 analyzes the results, specifically including common method biases, descriptive statistical analysis, validating factor analysis, and hypothesis testing. The Section 5 is a discussion of the theoretical implications, practical implications, and limitations and identifies possible future research directions.

## 2. Theoretical Background and Hypotheses Development

### 2.1. Threat–Rigidity Theory: The Theoretical Foundation

According to threat–rigidity theory, individuals exhibit cognitive and behavioral rigidity when they perceive a situation as threatening (Staw et al., 1981). Ambivalence toward digital–AI transformation—characterized by simultaneous positive and negative reactions—can be perceived as a psychological threat. This perceived threat triggers stress responses, narrowing employee focus to avoiding losses rather than pursuing gains, thereby reducing flexibility in decision-making and proactive behaviors like taking charge (Staw et al., 1981; Van Hootegem et al., 2019). This theoretical lens provides a foundation for understanding how ambivalence shapes employees’ responses to digital–AI transformation.

### 2.2. Ambivalence Toward Digital–AI Transformation

Ambivalence is a psychological state involving both positive and negative reactions to an object (Wu et al., 2024). It can create discomfort, affect emotions, and influence cognitive processing and decision-making (Meyerson & Scully, 1995). Employees’ acceptance of organizational change depends heavily on their perception of change legitimacy (Oreg & Sverdlik, 2011; Liang et al., 2024). Ambivalent employees engage in deliberate, risk-averse information processing (Maio et al., 1996; Cunningham et al., 2003), balancing opportunities (e.g., efficiency gains) and challenges (e.g., skill obsolescence) (Oreg & Sverdlik, 2011). While this balanced perspective aids strategic adaptation, it may also inhibit the bold, proactive actions essential for digital–AI success.

Moreover, digital–AI transformation transcends technological adoption—it demands fundamental shifts in mindset and organizational practices (Montero Guerra & Danvila-Del Valle, 2024). Despite the macro-level focus in prior research (Hanelt et al., 2021), the micro-level impact on employees remains underexplored (Wu et al., 2024). Recent studies highlight employees’ cognitive struggles, such as acceptance dilemmas (Trenerry et al., 2021) and meaning reconstruction (Van Der Schaft et al., 2024), underscoring the need to examine ambivalence as a key barrier to proactive change.

To address this gap, organizations must recognize and manage ambivalence effectively. In this way, they can transform the tension created by ambivalence into valuable strategic insights (Oreg & Sverdlik, 2011). However, unresolved ambivalence risks entrenching rigidity, as employees prioritize self-protection over organizational innovation (Staw et al., 1981). This study bridges this gap by investigating how ambivalence shapes taking-charge behavior through psychological mechanisms.

### 2.3. Ambivalence Toward Digital–AI Transformation and Taking-Charge Behavior

Taking-charge behavior refers to employees’ voluntary efforts to initiate change and improve organizational effectiveness (Moon et al., 2008; Fuller et al., 2012). Ambivalence, involving both positive and negative reactions, introduces uncertainty and can hinder proactive behaviors like taking charge. For example, employees uncertain about the impact of digital–AI transformation may hesitate to challenge the status quo, fearing unintended consequences (Muurlink et al., 2012). According to threat–rigidity theory, ambivalence amplifies perceived threats, leading to cognitive rigidity and risk-averse behaviors (Staw et al., 1981; Van Hootegem et al., 2019). This heightened threat perception can cause employees to prioritize short-term stability over long-term benefits, reducing their likelihood of taking charge.

Taking-charge behavior is an advanced form of proactive work behavior, characterized by voluntariness, spontaneity, and a focus on promoting organizational change and enhancement (Moon et al., 2008; Fuller et al., 2012). It requires employees to challenge the status quo, engage in innovative exploration, and balance strategic planning with practical action (Fuller et al., 2012; Love & Dustin, 2014). This behavior is driven by a deep concern for the organization’s future development and aims to drive continuous progress by strengthening organizational functions and improving operational efficiency (Choi, 2007).

Previous research determined that employee perceptions significantly predict proactive change behavior (Dysvik et al., 2016; Yan et al., 2021). Positive perceptions encourage proactive behaviors, while negative perceptions have the opposite effect (Yang et al., 2019; D. Liu et al., 2021). In the context of digital–AI transformation, employees may perceive a gap between their skills and transformation needs, feel their job stability is threatened, or find it difficult to adapt to the new work model. These perceptions can lead to subjective threat perception (Wu et al., 2024; Montero Guerra & Danvila-Del Valle, 2024). Research has confirmed that higher perceived threat levels are associated with negative coping strategies, such as avoidance behaviors (Beaudry & Pinsonneault, 2010; Butts et al., 2015). When employees perceive digital–AI technologies as a threat, they may experience negative emotions and cognitive rigidity, becoming more focused on the challenges and risks rather than the long-term benefits (Staw et al., 1981; Muurlink et al., 2012). This aligns with threat–rigidity theory, which suggests that cognitive limitations can lead to rigid behavioral patterns, making employees risk-averse and reluctant to try innovations (Muurlink et al., 2012).

**Hypothesis 1** **(H1).** *Ambivalence toward digital–AI transformation is positively associated with taking-charge behavior.*

### 2.4. The Dual Mediating Role of Job Engagement and Future Work Self-Salience

#### 2.4.1. The Mediating Role of Job Engagement

Threat–rigidity theory provides a critical lens for understanding how ambivalence toward digital–AI transformation triggers cognitive and behavioral rigidity (Staw et al., 1981). This theory posits that when employees perceive organizational change as a threat (e.g., role obsolescence and skill redundancy), they tend to narrow their cognitive processing, restrict information search, and revert to routine behaviors as a defensive mechanism (Staw et al., 1981; H. Lin et al., 2024). This theoretical framework is particularly salient in explaining the dual nature of digital–AI ambivalence: While employees may cognitively recognize the transformation’s benefits (e.g., efficiency gains, career growth), the simultaneous perception of threats (e.g., skill obsolescence, role ambiguity) activates the core mechanisms of threat–rigidity, cognitive constriction, and risk aversion (Wu et al., 2024; Staw et al., 1981).

Threat–rigidity theory posits that under perceived threat, employees exhibit cognitive rigidity, reducing their engagement in proactive behaviors (Staw et al., 1981). Ambivalence toward digital–AI transformation, as a subjective threat perception triggered by the external environment, may provoke a series of stress reactions. On the one hand, employees may recognize the necessity and potential benefits of digital–AI transformation, such as improving work efficiency and expanding career development space; on the other hand, they may be concerned about the uncertainties and challenges posed by the transformation, such as the pressure to update work skills and changes in work roles (Wu et al., 2024). This enhanced subjective threat perception can lead to psychological stress and cognitive rigidity, prompting employees to over worry about the challenges and risks and neglect the long-term benefits of the project (Staw et al., 1981). According to threat–rigidity theory, this cognitive limitation may lead to employee avoidance and conservative tendencies (Staw et al., 1981), thus avoiding risks and reducing job engagement.

Empirical research has shown that ambivalence significantly impacts employee job engagement (Yao et al., 2010; Han & Sears, 2024; Kaltiainen et al., 2020; Vakola et al., 2021). Studies have demonstrated that ambivalent employees experience heightened stress reactivity (Yao et al., 2010), which threat–rigidity theory attributes to the depletion of cognitive resources under perceived threats (Staw et al., 1981). For instance, one study found that LMX (Leader-Member Exchange) ambivalence is significantly correlated with job engagement and emotional exhaustion. Specifically, ambivalence negatively affects job engagement, which in turn hampers employees’ ability to adapt to transitions (Han & Sears, 2024). Additionally, Vakola et al. (2021) demonstrated that job engagement and job crafting mediate the relationship between ambivalence and adaptive or non-adaptive behaviors. Therefore, it is reasonable to infer that when employees are ambivalent about digital–AI transformation, their job engagement is likely to be inhibited.

Job engagement refers to the employee’s degree of active engagement and concentration on their work (Rich et al., 2010). Higher job engagement and work energy help employees to focus their attention, broaden their points of interest, and devote themselves to their job with a full mental state, providing important conditions for the formation of proactive taking-charge behavior (J. T. Chen et al., 2020; Zhou et al., 2020).

When exploring benevolent leadership and proactive change behavior, some scholars have found that job engagement plays a fully mediating role between the two, which implies that job engagement positively affects proactive change behavior (Q. Xu et al., 2018). Furthermore, several studies have confirmed, in different contexts, that job engagement is positively related to responsible employee behavior (Sun et al., 2022; S. L. Li et al., 2019). It can be inferred that employee job engagement is a crucial predictor of taking-charge behavior. When employees are actively engaged in their jobs, they are more likely to take responsibility proactively and contribute to the development of the organization. In summary, ambivalence toward digital–AI transformation may hinder employee job engagement, which in turn negatively influences their proactive taking-charge behavior. Therefore, work engagement may play a mediating role between ambivalence toward digital–AI transformation and taking-charge behavior.

**Hypothesis 2** **(H2).** *Ambivalence toward digital–AI transformation has a negative indirect effect on taking-charge behavior through job engagement.*

#### 2.4.2. The Mediating Role of Future Work Self-Salience

Threat–rigidity theory suggests that perceived threats activate two key mechanisms: (1) cognitive narrowing that prioritizes immediate threat resolution over long-term planning, and (2) resource conservation strategies that reduce investment in identity development activities (Staw et al., 1981). In the context of digital–AI ambivalence, employees’ conflicting evaluations (opportunity vs. existential threat) create precisely the type of “survival threat” that triggers these defensive processes (H. Lin et al., 2024).

As AI technology continues to evolve, employees’ anxiety and stress rise. Although they generally recognize the benefits of digital intelligence tools for their work, they are also deeply worried that AI may eventually replace their positions (Frey & Osborne, 2017), which maps their uncertainty and panic about the future development of their careers (Yin et al., 2024). On the one hand, the substitution risk latent in the introduction of AI in organizations is quietly shaking employees’ beliefs about the importance and sustainability of their own roles (Wu & Zhang, 2024); on the other hand, in the face of these perceived threats, they may feel powerless to resist and consequently perceive that their current jobs are in jeopardy, which negatively impacts their career prospects (H. Lin et al., 2024). These factors intertwine to produce ambivalence toward digital–AI transformation.

In the face of external stimuli, an individual’s behavioral response is based on their understanding and judgment (Martinko et al., 2002). Future work self-salience (FWSS) is an important cognitive indicator of the clarity of an employee’s future career plans (Y. Xu et al., 2019). Combined with threat–rigidity theory (Staw et al., 1981), ambivalence toward digital–AI transformation may trigger an employee’s cognitive assessment process, which in turn ripples through their future work self-salience. Considering the specificity of ambivalence in digital–AI transformation (Staw et al., 1981), this ambiguity and uncertainty can permeate employee perceptions of their future career prospects, which ultimately leads to ambivalence toward digital–AI transformation weakening their future work self-salience.

Future work self-salience motivates employees to pursue their career development (H. Lin et al., 2024). A clear possible self motivates individuals to generate actions in the direction that this self indicates (Leondari et al., 1998). Oyserman and Markus (1990) proposed that the possible self is a source of motivation that individuals use to control and guide their actions, inspiring them to act to generate change or achieve personal development (Hoyle & Sherrill, 2006). Consistent with this, the future work self also provides a source of motivation for individual action. Employee self-goal setting and achievement goal orientation not only direct them to respond proactively and increase work motivation but also help them to focus their attention, reduce the risks and threats in the process of proactive charge taking, and promote the implementation of taking-charge behavior (Bandura, 1991).

When ambivalence is framed as a threat signal, three cascading effects emerge: Threatened employees disproportionately focus on immediate career risks (e.g., AI substitution) at the expense of long-term professional identity construction (Hall et al., 1987). Cognitive rigidity manifests as reduced mental simulations of future professional selves—a core component of future work self-salience (FWSS) (Y. Xu et al., 2019). Conservation of cognitive resources leads to the diminished exploration of potential future identities (Staw et al., 1981), creating a self-fulfilling prophecy where limited identity investment exacerbates perceived career threats (Karoly, 1993).

Empirical evidence supports this theoretical pathway. Research on technological disruption demonstrates that employees perceiving AI as a job threat show 37% lower engagement in future skill development activities—a behavioral manifestation of threatened FWSS (Frey & Osborne, 2017). Crucially, this aligns with threat–rigidity theory’s prediction that threatened individuals prioritize routine identity maintenance over adaptive identity growth (Staw et al., 1981). This theoretical integration explains why ambivalent employees struggle to maintain clear FWSS. As per the theory’s escalation cycle (Staw et al., 1981), ambivalence-induced stress overload depletes the cognitive resources required for complex future self-construal (May et al., 2004), and perceived career instability activates loss-prevention motivation, shifting the focus from “becoming” (future-oriented) to “preserving” (present-focused) (Zhao & Liu, 2024). Accordingly, the following hypothesis is proposed.

**Hypothesis 3** **(H3).** *Ambivalence toward digital–AI transformation has a negative indirect effect on taking-charge behavior through future work self-salience.*

### 2.5. The Chained Mediating Effect of Future Work Self-Salience and Job Engagement

When ambivalence is perceived as a career threat, threat–rigidity theory predicts a cascading rigidity effect: “cognitive constriction→identity destabilization→diminished engagement→behavioral withdrawal” (Staw et al., 1981; H. Lin et al., 2024). This chain mechanism delineates how diminished future work self-salience (FWSS) and job engagement jointly transmit the detrimental effects of ambivalence.

Stage 1: From Ambivalence to FWSS Attenuation

Through the lens of threat–rigidity theory, digital–AI ambivalence activates defensive cognitive simplification (Staw et al., 1981) in three ways: (1) Threat Priming: Conflicting evaluations (opportunity vs. job threat) trigger survival-mode cognition, prioritizing immediate risk mitigation over long-term identity construction (Martinko et al., 2002). (2) Temporal Discounting: Employees overweight near-term career risks (e.g., AI substitution) while underweighting future professional growth potential (W. Lin et al., 2016; Hall et al., 1987; Karoly, 1993). (3) Identity Compression: Cognitive resources are diverted from future self-imagery to threat monitoring, constraining the mental “bandwidth” for FWSS development (May et al., 2004).

Stage 2: From FWSS to Job Engagement

Threat–rigidity theory further explains how FWSS depletion affects job engagement through goal-system disintegration (Staw et al., 1981) in the following ways: (1) Directionality Loss: Blurred future selves deprive employees of the “cognitive compass” needed to align daily tasks with aspirational goals (W. Lin et al., 2016; May et al., 2004); (2) Energy Conservation: Threatened individuals instinctively preserve resources by reducing discretionary effort expenditure—the behavioral core of job engagement (Staw et al., 1981; H. Lin et al., 2024); (3) Feedback Amplification: Lower engagement limits skill development opportunities, further eroding FWSS clarity in a self-reinforcing loop (Staw et al., 1981; Hall et al., 1987). Specifically, future work self-salience helps individuals recognize the gap between their current state and their future goals. Compared to vague goal setting, a clear future work self-setting can drive them to effectively engage their energies and resources in goal pursuit, facilitating higher outputs (Hall et al., 1987; Karoly, 1993). This implies that future work self-salience may be closely related to job engagement. Some scholars have preliminarily verified the positive effect of future work self-salience on job engagement (W. Lin et al., 2016), partly validating the theory’s prediction of threat-propagation mechanisms.

Stage 3: Cumulative Rigidity Effects

The chained mediation embodies threat–rigidity theory’s escalation principle (Staw et al., 1981). Several studies have confirmed, in different contexts, that job engagement is positively related to responsible employee behavior (Sun et al., 2022; S. L. Li et al., 2019). Job engagement is a crucial predictor of taking-charge behavior. Based on threat–rigidity theory (Staw et al., 1981), the ambivalence caused by digital–AI transformation may lead to a reduction in employees’ future work self-salience, and the ambiguous goal is likely to trigger rigidity at the cognitive level. This then manifests itself as a reduction in job engagement, i.e., state rigidity, and ultimately hinders individuals’ taking-charge behaviors. This theoretical progression explains why isolated interventions (e.g., enhancing engagement without addressing FWSS) often fail in digital transformations—they neglect the self-reinforcing nature of threat–rigidity cycles. Based on this, we propose the following hypothesis.

**Hypothesis 4** **(H4).** *Future work self-salience and job engagement jointly play a chained mediating role in the negative effect of ambivalence toward digital–AI transformation on taking-charge behavior.*

**Figure 1:**
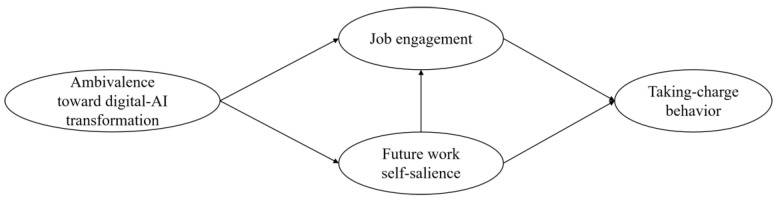
Research model.

In summary, this study constructed a model (see Figure 1) to explore how ambivalence toward digital–AI transformation affects taking-charge behavior based on threat–rigidity theory.

## 3. Methodology

### 3.1. Measures

The questionnaire in this study consists of two modules: demographic information and main content. The demographic module covers gender, age, education, and rank. The main content module contains four sections: the independent variable (ambivalence toward digital–AI transformation), the mediating variables (job engagement, future work self-salience), and the dependent variable (taking-charge behavior). All variables were measured using a 7-point Likert scale (from 1 (strongly disagree) to 7 (strongly agree)) because this scale not only provides high resolution and sensitivity but also captures workers’ psychological states and behavioral tendencies in more detail, thereby increasing the accuracy and reliability of the data (Zhao & Liu, 2024). To enhance the adaptability of the scales, the following measures were taken: first, the scales were translated from English to Chinese by professional translators and underwent several rounds of proofreading to ensure semantic accuracy. Second, all formal surveys were conducted in Chinese to ensure that respondents understood the questions accurately and provided honest and reliable feedback. These steps significantly improved the adaptability of the scale and ensured the accuracy and reliability of the survey results (Ho, 2024).

Ambivalence toward digital–AI transformation. We used a four-item scale from previous studies (Oreg & Sverdlik, 2011) that had been widely adopted in researching employees’ attitudes toward organizational changes, including statements such as “When I think about the digital–AI transformation of the company I experience both good and bad feelings”. The scale’s Cronbach’s alpha was 0.898, indicating its reliability in measuring this construct.

Job Engagement. We drew on the work engagement scale developed by Rich et al. and other scholars (Rich et al., 2010), which had been extensively validated in numerous work-related attitude and behavior studies, and adapted it as a whole to improve this study’s efficiency and usefulness (Farquhar, 1990). The composite score was calculated by averaging the six items as suggested by Schaufeli et al. (2006). These included statements such as “At work, I concentrate on my job”. Cronbach’s alpha was 0.797.

Future work self-salience. We measured future work self-salience using five items widely adapted from a previous study (Strauss et al., 2012), for example, “The mental picture of this future is very clear”. The Cronbach’s alpha was 0.882.

Taking-charge behavior. Taking-charge behavior was assessed using five items adapted from Klein et al.’s proactive organizational behavior scale (Klein et al., 2025), which had been used in many previous studies on proactive organizational behaviors. An example is “I bring about improved procedures for the work unit or department”. The Cronbach’s alpha was 0.816.

Control variables. It has been shown that employees’ proactive change behaviors are affected by demographic characteristic variables such as gender, age, education, and rank (R. Li et al., 2016), so we selected these variables as control variables.

### 3.2. Participants and Procedure

Data were collected via the Credamo platform, whose strict participant screening protocol ensures that working professionals have digital–AI conversion experience, and that recruited participants have the most accurate attention and comprehension checks (Del Ponte et al., 2024). It was selected as a data collection platform in recent AI-related research (H. Chen et al., 2025).

A series of screening procedures were carried out. Firstly, drawing on previous studies, the definition of “digital–AI transformation” was clearly explained to the respondents prior to providing the questionnaire (P. Liu et al., 2024). During the initial screening stage, the respondents were explicitly informed of the eligibility criteria: “First, the company you are working for is currently undergoing or has already completed digital-intelligent transformation. Please ensure that you meet our survey requirements”. “Second, your work involves interaction with technologies such as big data and artificial intelligence”. To ensure that the respondents read these conditions, an attention screening question was asked to eliminate those who were not careful.

Subsequently, the respondents could begin the formal questionnaire. Besides providing the demographic information (control variables), ambivalence toward digital–AI transformation, and other main variables, a fill-in-the-blank question was also asked, which read, “Do you have any other ambivalent thoughts about your company’s digital–AI transformation? If so, please briefly describe them here (1–2 sentences)”. The purpose of this question was to focus the respondents on answering the additional questions. The survey was conducted on 13 November 2024. We distributed 450 questionnaires and retrieved 343 valid responses, excluding 107 due to ineligibility or failing attention checks. The valid response rate was 76.2%. The demographic characteristics of the respondents are shown in Table 1.

## 4. Results

The theoretical model was analyzed using SPSS v26.0 (IBM Corp., Armonk, NY, USA), AMOS v26.0 (IBM Corp., Armonk, NY, USA), and PROCESS v4.1 (Andrew F. Hayes, Chapel Hill, NC, USA). First, descriptive analyses, including mean, standard deviation, and correlation analyses, were conducted for ambivalence toward digital–AI transformation and other main variables. Second, CFA was used to verify the fit of the factor structure of the measurement instrument to the theoretical model. Finally, SPSS and PROCESS were used to test the hypotheses and analyze the main, mediating, and moderating effects.

### 4.1. Common Method Bias

We used the common method bias test, which showed that four factors with characteristic roots greater than 1 were obtained without rotation. Harman’s single-factor test indicated that the first factor explained only 33.837% of variance, below the 40% threshold, suggesting no severe common method bias.

### 4.2. Descriptive Statistical Analysis

Table 2 presents the results of the descriptive statistical analysis. First of all, the data stability was good and there was no standard deviation greater than the mean (Gao & Gao, 2024). According to the table, the square root of the constructed AVE values was greater than the correlation coefficient between these variables to obtain sufficient discriminant validity. According to the correlation results, ambivalence toward digital transformation was negatively correlated with taking-charge behavior (r = −0.141, *p* < 0.01), job engagement (r = −0.153, *p* < 0.01), and future work self-salience (r = −0.268, *p* < 0.01). In addition, future work self-salience was positively related to job engagement (r = 0.514, *p* < 0.01) and taking-charge behavior (r = 0.507, *p* < 0.01). Job engagement was positively correlated with taking-charge behavior (r = 0.617, *p* < 0.01). All the hypotheses of this study were preliminarily supported.

### 4.3. Confirmatory Factor Analysis

In this study, a confirmatory factor analysis was used for ambivalence toward the digital-AI transformation, job engagement, future work self-salience, and taking-charge behavior to test the discriminatory validity results between variables (see Table 3). Compared with alternative models, the four-factor model presented the best fitting indices (χ^2^/df = 1.832 < 3; TLI = 0.950 > 0.90; CFI = 0.957 > 0.90; RMSEA = 0.049 < 0.08), indicating that the four-factor model had good discriminant validity.

### 4.4. Hypothesis Testing

**Figure 2:**
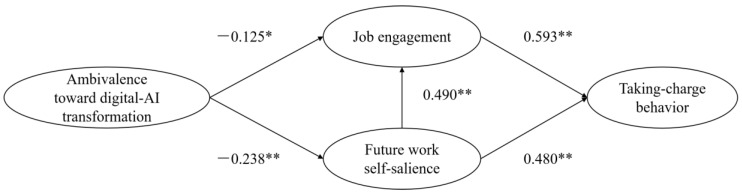
Resulting model. Notes: * *p* < 0.05, ** *p* < 0.01.

As shown in Table 4, ambivalence toward the digital-AI transformation had a negative and significant effect on proactive taking-charge behavior (β = −0.112; *p* < 0.05), and H1 was supported. This suggests that when employees experience mixed feelings about digital transformation, they are less likely to take the initiative in driving workplace changes, highlighting the importance of addressing employee ambivalence to foster proactive behaviors during organizational transitions. As shown in Figure 2, ambivalence toward digital–AI transformation had a significant negative effect on job engagement (β = −0.125; *p* < 0.05); job engagement had a significant positive effect on taking-charge behavior (β = 0.593; *p* < 0.01), and H2 was supported. This indicates that employees’ uncertainty about digital–AI transformation reduces their engagement at work, which in turn diminishes their willingness to take charge. Organizations should focus on enhancing job engagement to mitigate the negative impact of ambivalence on proactive behaviors. Ambivalence toward digital–AI transformation had a significant negative effect on future work self-salience (β = −0.238; *p* < 0.01); job engagement had a significant positive effect on taking-charge behavior (β = 0.480; *p* < 0.01), and H3 was supported. This implies that employees’ mixed feelings about digital–AI transformation weaken their sense of future work identity, which further reduces their engagement and proactive behaviors. Strengthening an employee’s vision of their future role in the transformed workplace could help counteract these effects.

For future work self-salience, the effect size (R^2^) was 0.12. This indicates that ambivalence toward digital–AI transformation and demographic factors are meaningful predictors of how employees perceive their future roles. Organizations should provide clear communication and training to help employees envision their future roles in the transformed workplace, thereby reducing uncertainty and enhancing their sense of purpose. For job engagement, the effect size (R^2^) was 0.29, suggesting that future work self-salience and ambivalence toward digital–AI transformation are significant predictors of employees’ engagement at work. This highlights the importance of improving the clarity of their future roles in fostering higher engagement during organizational change. To counterbalance the negative effects of ambivalence, organizations should focus on strengthening employees’ future work identity through career development programs and role clarity initiatives. For taking-charge behavior, the effect size (R^2^) was 0.44, indicating that job engagement, future work self-salience, and ambivalence toward digital–AI transformation are strong predictors of proactive behavior. This underscores the combined role of psychological and contextual factors in driving employees’ willingness to take the initiative during digital transformation. Organizations should prioritize interventions that enhance job engagement and future work self-salience, such as fostering a supportive work environment and providing opportunities for skill development. For the total effect model of taking-charge behavior, the effect size (R^2^) was 0.08. This suggests that ambivalence toward digital–AI transformation has a direct but limited impact on proactive behavior, with most of its influence mediated through job engagement and future work self-salience. This emphasizes the importance of addressing both engagement and future role clarity to mitigate the negative effects of ambivalence on proactive behaviors.

In addition, we conducted a mediation analysis (see Table 5) to assess the mediating role of job engagement and future work self-salience in the relationship between ambivalence toward digital–AI transformation and taking-charge behavior. When job engagement was added to the model, the effect of ambivalence toward digital–AI transformation on taking-charge behavior became non-significant, whereas the indirect effect through job engagement was significant (β = −0.045; *p* < 0.01). This result reveals that job engagement fully mediates the relationship between ambivalence toward digital–AI transformation and taking-charge behavior, thus validating H2. This result suggests that job engagement fully mediates the relationship between ambivalence and taking-charge behavior, emphasizing the critical role of engagement in translating employees’ attitudes into proactive actions during digital–AI transformation. Similarly, when future work self-salience was added to the model, the effect of ambivalence toward digital–AI transformation on taking-charge behavior became non-significant, whereas the indirect effect through future work self-salience was significant (β = −0.069; *p* < 0.01). This finding underscores that an employee’s perception of their future role in the transformed workplace plays a pivotal role in shaping their proactive behaviors, highlighting the need for organizations to clarify and reinforce employees’ future work identities during digital–AI transitions. This result suggests that future work self-salience fully mediates the relationship between ambivalence toward digital–AI transformation and taking-charge behavior, thus validating H3. Finally, the chain-mediated effect of future work self-salience and job engagement holds (β = −0.033; *p* < 0.01), and H4 is validated. This chain mediation effect reveals that employees’ ambivalence toward digital–AI transformation not only directly affects their engagement and future work identity but also indirectly influences their proactive behaviors through these mediators. Organizations should adopt a holistic approach to address both engagement and future work identity to foster proactive behaviors during digital transformation.

## 5. Discussion

### 5.1. Theoretical Implications

The present study offers several theoretical implications for understanding ambivalence toward digital–AI transformation and its impact on taking-charge behavior within the context of threat–rigidity theory. Firstly, it extends the application of threat–rigidity theory by demonstrating how subjective perceptions of threat, in the form of ambivalence, can lead to behavioral rigidity, specifically in the context of digital–AI transformation. While prior research has primarily focused on external threats (e.g., economic crises or competitive pressures) as triggers of rigidity (Yan et al., 2021), this study highlights ambivalence as an internal psychological threat that similarly inhibits proactive behaviors. This finding aligns with Yan et al. (2021) but extends their work by emphasizing the role of internal cognitive processes in shaping threat responses.

Secondly, this study provides a more nuanced view of ambivalence, recognizing it as a complex attitude comprising both positive and negative reactions toward digital–AI transformation. This dualistic perspective challenges the traditional binary approach to understanding employee reactions to change and offers a more comprehensive framework for examining the multifaceted nature of employee attitudes and behaviors during organizational transformations (Beaudry & Pinsonneault, 2010). Our findings are consistent with recent work on ambivalence in organizational contexts, which suggests that mixed emotions can coexist and influence behavior in unique ways. However, we extend this literature by demonstrating that ambivalence not only affects immediate reactions but also has downstream effects on proactive behaviors through psychological mediators.

Furthermore, this study extends threat–rigidity theory by demonstrating that ambivalence influences proactive behaviors through psychological mediators, namely, job engagement and future work self-salience. These findings highlight the importance of employee cognition in organizational change, as these mediators play a critical role in translating ambivalence into actionable behaviors (Frey & Osborne, 2017; Leondari et al., 1998). Our results thus contribute to a more refined understanding of the psychological processes that drive employee responses to digital–AI transformation.

### 5.2. Practical Implications

The findings of this study have significant practical implications for organizations undergoing digital–AI transformation. Firstly, by recognizing the negative impact of ambivalence on taking-charge behavior, organizations can develop targeted interventions to mitigate employee ambivalence. These may involve clear communication strategies that address both the opportunities and challenges associated with digital–AI transformation, thereby fostering a more balanced and informed perspective among employees (D. Liu et al., 2021). For example, organizations can implement regular town hall meetings or Q&A sessions where leaders transparently discuss the goals, benefits, and potential challenges of AI integration, helping employees feel more informed and less uncertain.

Secondly, this study highlights the importance of job engagement and future work self-salience in driving proactive work behaviors. Organizations can leverage this insight by creating environments that enhance job engagement, such as through meaningful work design, supportive leadership, and opportunities for skill development. Additionally, organizations can foster a strong sense of future work self-salience by providing clear career paths and development opportunities that align with the digital–AI transformation journey (Butts et al., 2015). For instance, companies can introduce mentorship programs that connect employees with AI-specialized roles, helping them visualize their future career paths and enhancing their sense of future work self-salience (Butts et al., 2015). Additionally, organizations can offer upskilling workshops or certifications in AI-related skills, empowering employees to actively participate in the transformation process.

Thirdly, this study suggests that organizations should consider the psychological well-being of employees during digital–AI transformations. By addressing the ambivalence that employees may feel, organizations can foster a more positive and proactive workforce, which in turn can lead to more effective organizational change and enhanced competitive advantage in the digital era (Rich et al., 2010). For example, organizations can establish employee resource groups (ERGs) focused on digital transformation, providing a safe space for employees to share concerns, ask questions, and collaborate on solutions. This can reduce feelings of isolation and build a sense of community during times of change.

### 5.3. Limitations and Future Research Directions

Firstly, the cross-sectional design limits the ability to draw causal inferences about the relationships between ambivalence, job engagement, future work self-salience, and taking-charge behavior. Future research could benefit from longitudinal designs to better understand the developmental trajectories of these constructs over time (Q. Xu et al., 2018).

Secondly, this study relies on self-reported data, which may be subject to common method bias. Future studies could incorporate objective performance metrics or multi-source feedback to validate the findings (Zhou et al., 2020).

Thirdly, while this study focuses on job engagement and future work self-salience as mediators, other potential mediators or moderators, such as organizational support or individual differences, could be explored in future research. This would provide a more comprehensive understanding of the factors that influence the relationship between ambivalence and taking-charge behavior in the digital–AI era (Han & Sears, 2024).

Finally, this study does not account for cultural or contextual factors that might influence ambivalence toward digital–AI transformation. Future research should examine how cultural differences shape ambivalence and its outcomes. Additionally, organizational cultures that prioritize innovation and adaptability may mitigate ambivalence more effectively than those with rigid structures. Additionally, social factors (e.g., generational differences, the stability of positions within the organizational hierarchy, the adaptability gained by experience) may further moderate these relationships. Exploring these contextual nuances would provide deeper insights into the boundary conditions of our findings and enhance their generalizability across diverse settings (P. Liu et al., 2024).

## 6. Conclusions

Digital–AI transformation poses a significant challenge to organizations and their employees, eliciting a range of complex psychological responses, among which ambivalence is particularly salient. This study investigates the impact of ambivalence toward digital–AI transformation on taking-charge behavior, drawing on threat–rigidity theory to explore the underlying mechanisms.

Firstly, this study confirms that ambivalence toward digital–AI transformation negatively influences taking-charge behavior, suggesting that employees who experience mixed feelings about such transformations are less likely to engage in proactive change behaviors. This finding underscores the importance of understanding and managing the ambivalence that employees may harbor during periods of technological and organizational change (Yan et al., 2021). While this study focuses on AI-driven change, the findings may extend to other forms of organizational transformation, such as automation in manufacturing or digitalization in service industries. For example, employees facing automation in manufacturing may similarly experience ambivalence, which could hinder their willingness to adopt new technologies or processes.

Secondly, this study reveals that job engagement and future work self-salience fully mediate the relationship between ambivalence toward digital–AI transformation and taking-charge behavior. This highlights the pivotal role of psychological engagement and a future-oriented self-concept in translating employees’ ambivalent attitudes into actionable behaviors. Employees with higher levels of job engagement and a more salient future work self are better equipped to proactively navigate the complexities of digital–AI transformation (Butts et al., 2015; Frey & Osborne, 2017). These mechanisms are likely applicable to other transformative contexts, such as the adoption of green technologies or organizational restructuring, where employee engagement and future-oriented thinking are equally critical for successful implementation.

Thirdly, the chained mediating effect of future work self-salience and job engagement was also confirmed, indicating that the interplay between these psychological states is crucial in the context of digital–AI transformation. This finding emphasizes the need for organizations to consider the interconnected nature of employee attitudes and responses during transformation processes (Oyserman & Markus, 1990). This insight is relevant beyond AI transformation, as similar psychological dynamics may arise in other high-stakes organizational changes, such as mergers and acquisitions or shifts toward remote work models.

Theoretically, this study contributes to the literature on organizational change and employee behavior by providing a more nuanced understanding of the role of ambivalence and its downstream effects on proactive work behaviors. Practically, it offers guidance for organizations on how to foster positive employee attitudes and behaviors that can facilitate successful digital–AI transformations (D. Liu et al., 2021). These recommendations are not limited to AI contexts; they can also inform strategies for managing employee responses to other types of organizational change, such as the introduction of new business models or large-scale process reengineering. Despite its contributions, this study is subject to limitations, including its cross-sectional design and reliance on self-reported data. Future research should address these limitations and further explore the boundary conditions and additional factors that may influence the relationship between ambivalence, job engagement, future work self-salience, and taking-charge behavior. For example, future studies could examine how cultural differences or industry-specific factors shape ambivalence and its outcomes across various transformation contexts. Nevertheless, this study provides a solid foundation for understanding the intricacies of employee ambivalence and its implications for organizational change in the digital–AI era (Q. Xu et al., 2018; S. L. Li et al., 2019).

## Figures and Tables

**Table 1:** Characteristics of survey respondents (n = 343).

Characteristics	Classification	Frequency	%
Gender	Male	229	33.2
	Female	114	66.8
Age	25 years old and below	62	18.1
	26–30	100	29.1
	31–40	150	43.7
	41–50	16	4.7
	51–60	15	4.4
	Over 60 years old	0	0.0
Education	College degree or below	19	5.5
	Bachelor’s degree	255	74.3
	Master’s degree	61	17.8
	PhD	8	2.4
Rank	Senior-level managers (Level 4)	23	6.7
	Middle-level managers (Level 3)	105	30.6
	Front-line supervisors (Level 2)	98	28.6
	Non-supervisory staff (Level 1)	117	34.1

**Table 2:** Mean, standard deviation, and correlations (n = 343).

Variables	M	SD	1	2	3	4	5	6	7	8
Gender	0.670	0.472								
Age	2.480	0.985	0.131 *							
Education	2.170	0.547	0.014	0.077						
Position	2.900	0.953	−0.087	−0.441 **	−0.276 **					
ADAT	4.394	1.359	0.073	−0.135 *	−0.061	0.101	**0.831**			
JE	5.846	0.637	0.061	0.230 **	0.001	−0.208 **	−0.153 **	**0.632**		
FWSS	5.280	1.002	−0.075	0.166 **	0.005	−0.220 **	−0.268 **	0.514 **	**0.775**	
TCB	5.510	0.825	0.051	0.189 **	0.065	−0.249 **	−0.141 **	0.617 **	0.507 **	**0.693**

Note. ADAT = ambivalence toward the digital-AI transformation; JE = job engagement; FWSS = future work self-salience; TCB = taking-charge behavior. Diagonal elements (in bold) are the square roots of the AVE of each construct. * *p* < 0.05, ** *p* < 0.01.

**Table 3:** Results of confirmatory factor analysis.

Factors	χ^2^/df	TLI	CFI	RMSEA
Four-factor model: ADAT, JE, FWSS, TCB	1.832	0.950	0.957	0.049
Three-factor model: ADAT, JE, FWSS + TCB	3.760	0.834	0.854	0.900
Two-factor model: ADAT, JE + FWSS + TCB	4.521	0.788	0.812	0.101
One-factor model: ADAT + JE + FWSS + TCB	9.044	0.516	0.567	0.153

Note. ADAT = ambivalence toward the digital-AI transformation; JE = job engagement; FWSS = future work self-salience; TCB = taking-charge behavior.

**Table 4:** Results of hypotheses.

Relationships	Path Coefficient (β)	Hypotheses	Results
ADAT→TCB	−0.112 *	H1	Supported
ADAT→JE	−0.125 *	H2	Supported
JE→TCB	0.593 **		
ADAT→FWSS	−0.238 **	H3	Supported
FWSS→TCB	0.480 **		

Notes: * *p* < 0.05, ** *p* < 0.01.

**Table 5:** Results of the mediating analyses.

Total Effect		Direct Effect		Indirect Effect		95% Confidence Interval	
Relationship	Path Coefficient (β)	Relationship	Path Coefficient (β)	Relationship	Path Coefficient (β)	Lower	Upper
ADAT→TCB	−0.068 *	ADAT→TCB	−0.024	ADAT→JE→TCB	−0.045 **	−0.083	−0.010
			0.001	ADAT→FWSS→TCB	−0.069 **	−0.102	−0.041
			0.004	ADAT→FWSS→JE→TCB	−0.033 **	−0.055	−0.016

Notes: * *p* < 0.05, ** *p* < 0.01.

## Data Availability

The data presented in this study are available on request from the corresponding author.
